# MicroRNA-424 inhibits Akt3/E2F3 axis and tumor growth in hepatocellular carcinoma

**DOI:** 10.18632/oncotarget.4811

**Published:** 2015-08-03

**Authors:** Hao Yang, Wei Zheng, Xiao Shuai, Rui-Min Chang, Lei Yu, Feng Fang, Lian-Yue Yang

**Affiliations:** ^1^ Liver Cancer Laboratory, Xiangya Hospital, Central South University, Changsha 410008, Hunan, China; ^2^ Department of Geratic Surgery, Xiangya Hospital, Central South University, Changsha 410008, Hunan, China; ^3^ Department of Surgery, Xiangya Hospital, Central South University, Changsha 410008, Hunan, China

**Keywords:** hepatocellular carcinoma, miR-424, Akt3, E2F3, tumor growth

## Abstract

By comparing the expression profiles of miRNAs in different subtypes of HCC, we identified miR-424 as a HCC related miRNA. We found that the expression of miR-424 was significantly decreased in HCC tissues and six liver cancer cell lines. Significantly, its expression levels were correlated with tumor size, multiple nodules, vein invasion, TNM stage and overall survival of HCC. We showed that up-regulated miR-424 suppressed HCC cell proliferation *in vivo* and *in vitro*. Multi-pathway reporter arrays suggested that miR-424 suppressed the pRb-E2F pathway. Consistently, Akt3 and E2F3 were identified as the targets of miR-424 as evidenced by that ectopic miR-424 expression suppressed Akt3 and E2F3 expressions. Silencing Akt3 and E2F3 by siRNA pheno-copied the effect of ectopic miR-424 on HCC growth. Whereas, overexpression of Akt3 and E2F3 attenuated the effect of miR-424 on HCC growth. Together, our data demonstrated a tumor suppressor role for miR-424 in HCC development and progression with therapeutic implications. The strong correlation of miR-424 expression with HCC patient survival suggests that miR-424 could be a valuable biomarker for HCC prognosis.

## INTRODUCTION

Hepatocellular carcinoma (HCC) is one of the most common human cancers in the world. It ranks as the fifth most common malignancy and the second leading cause of cancer-induced death in men and the sixth in women over the world, resulting in more than 695, 900 deaths happened each year [1, 2]. Although its mortality decreased along with the advancement of surgical resection, the long-term survival remains unsatisfactory. It is reported that the 5-year survival rate is only 20 to 30% in HCC patients after surgical resection [3, 4], mainly due to the high recurrence rate of HCC. Therefore, it is an urgent issue to understand the mechanisms that contribute to HCC survival and growth.

Previously, we found a specific subtype of HCC that was only around 5 cm in diameter with a single lesion but grew expansively within an intact capsule or pseudocapsule. In addition, the tumor possessed unique clinical and pathological characteristics and indicated good prognosis for patients after surgery [4]. We categorized this special HCC as solitary large hepatocellular carcinoma (SLHCC). Although much work had been done [5–10], the molecular characteristics of SLHCC are still elusive.

MicroRNAs (miRNAs) are a class of small, endogenously expressed, well-conserved noncoding RNA molecules with 18–25 nucleotides (nt). It has been recognized that aberrantly expressed miRNAs play essential roles in a variety of biological processes [11]. Given that more than 50% of miRNAs are located in cancer-associated genomic regions or in fragile sites, miRNAs may play an important role in cancer pathogenesis [12]. Indeed, it has been demonstrated that aberrant miRNA expression contributes to carcinogenesis and cancer development by promoting oncogene expression or by inhibiting tumor suppressor genes in HCC [13–17]. Previously, we found that SLHCC had a unique miRNA profile and aberrant miRNAs expression contributed to special molecular characteristics and HCC survival [5, 17]. However, more work need to be done to reveal the role of miRNAs in HCC.

In present study, we profiled miRNA expressions by analysis of 840 mammalian miRNAs in hepatocellular carcinoma from 30 HCC samples. miR-424 was identified to be significantly down-regulated in HCC tissues as compared with that of adjacent nontumorous liver tissues (ANLTs). Interestingly, miR-424 displayed similar expression levels between SLHCC and SHCC (small HCC, tumor ≤ 5 cm), but much lower levels in NHCC (nodular HCC, node number ≥ 2). These results were further confirmed by quantitative real-time PCR in 96 paired cases of human HCC tissues and cell lines. Significantly, we found that HCC patients with high miR-424 expressions had much longer overall survival time and disease-free survival time than those with low miR-424 expressions. These results were obtained from two different HCC patients cohort (training cohort and validation cohort). We identified miR-424 suppresses HCC cell proliferation by targeting Akt3 and E2F3. In summary, we have combined experimental and clinical studies to establish MicroRNA-424/Akt3/E2F3 axis functions as tumor suppressor in HCC growth.

## RESULTS

### miR-424 is down-regulated in HCC tissues and its expression level is Inversely correlated with Ki-67

**Figure 1:**
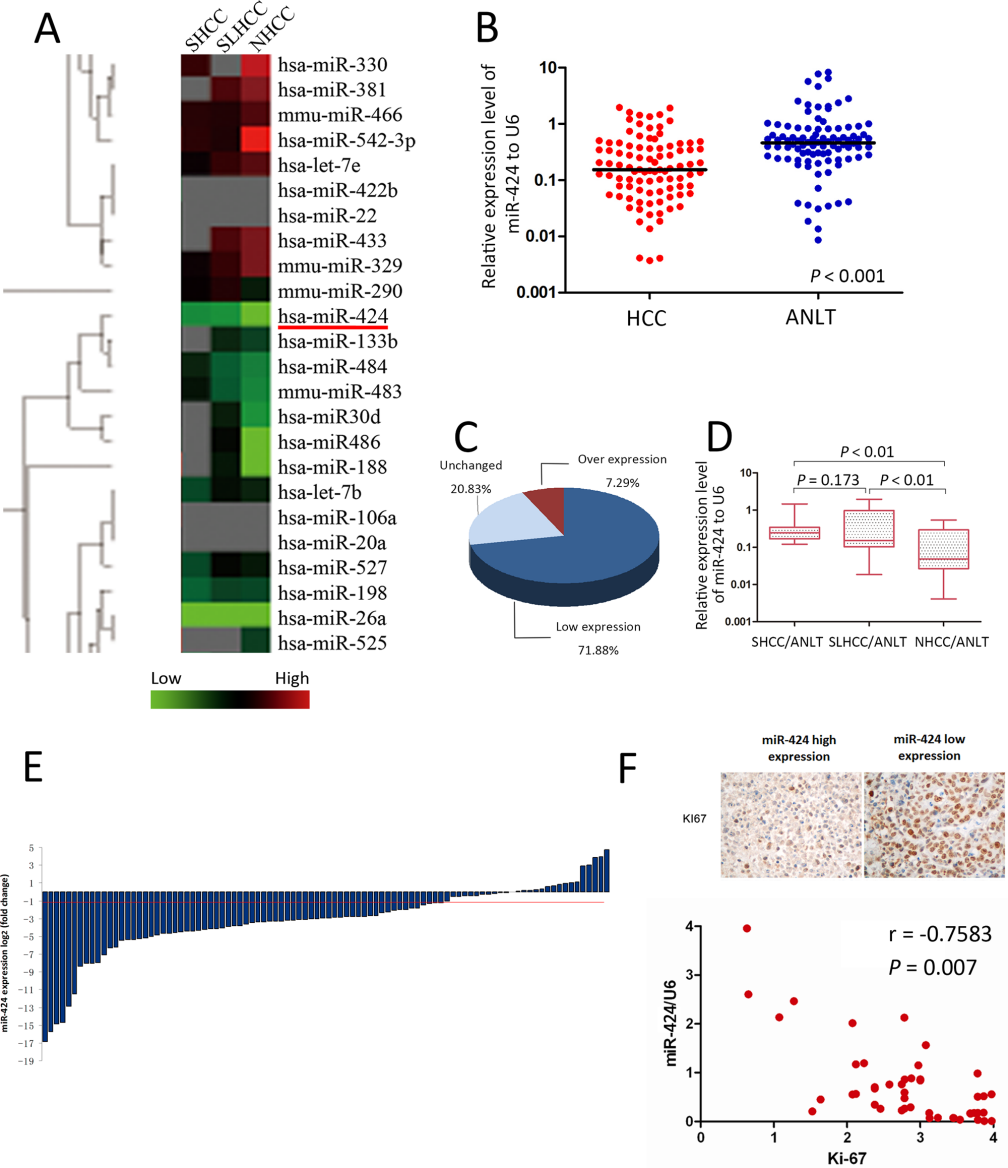
miR-424 is frequently down-regulated in HCC and its expression level is inversely correlated with Ki-67 **A.** Cluster analysis of miRNAs expression profiles of SHCC, SLHCC and NHCC *vs*. ANLTs. Overexpression is indicated with red, whereas underexpression is coded with green. **B.** The expression of miR-424 was three times lower in HCC tissues as compared with that of ANLTs **C & E.** Expression of miR-424 in 96 pairs of HCC tissues and the corresponding ANLTs. Expression levels of miR-424 were normalized to the corresponding levels of U6 snRNA. Data were analyzed using a ΔΔCt approach and expressed as log 2 fold change (ΔΔCt [HCC/ANLT]). **D.** miR-424 expression in SHCC, SLHCC and NHCC was calculated by a ΔΔCt approach and expressed as log2 fold change (ΔΔCt [HCC/ANLT]). The expression level was compared with independt *t* test. **F.** miR-424 expression level is inversely correlated with Ki-67 (*r* = −0.7583, *P* = 0.007).

The miRNA microarray analysis revealed that miR-424 was significantly down-regulated in all three subtypes of HCC tissues. Moreover, the expression of miR-424 was much lower in NHCC than that in SLHCC and SHCC (Fig. 1A). To confirm this result, real-time quantitative PCR was performed in 96 cases of HCC tissues and ANLTs. In general, a 3.0 fold decrease for miR-424 expression was noted in HCC tissues as compared with that of ANLTs (Fig. 1B). Comparative analysis of paired HCCs with ANLTs further revealed that reduced miR-424 expression (more than two-fold [i.e., log2 (fold change) < −1]) was observed in 69 (71.88%) cases, suggesting that reduction of miR-424 was a frequent event in human HCC (Fig. 1C&1E). We further analyzed the expression differences of miR-424 in three subtypes of HCC (SHCC, SLHCC and NHCC). Consistent with the miRNA array data, median miR-424 expression levels were similar in SLHCC and SHCC but much lower in NHCC (0.193 vs. 0.048, *P* < 0.001 and 0.244 vs. 0.048, *P* < 0.001; Fig. 1D). These results were further confirmed by a totally different validation cohort which contained 70 cases of matched fresh HCC specimens and ANLTs. In this validation cohort, the expression of miR-424 was down-regulated in 73% HCC patients and only 3% was up-regulated (Figure S1). Given that the expression of the human Ki-67 protein is strictly associated with cell proliferation and frequently used as a proliferation biomarker [18, 19], we detected the expression of Ki-67 in these HCC tissues by IHC (Fig. 1F). We found that the expression of miR-424 was inversely correlated with Ki-67 (*r* = −0.758, *P* < 0.001; Table S1, Fig. 1F). These results suggested that miR-424 might play as a suppressor in HCC growth and progression.

### Down-regulation of miR-424 is associated with HCC poor prognosis

**Figure 2:**
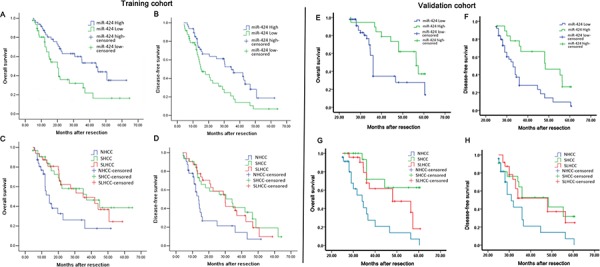
Survival relevance analysis of miR-424 expression in two HCC patients cohorts (A–D: Training cohort, E–H: Validation cohort) **A, B & E, F.** Survival relevance analysis of miR-424 expression in HCCs. According to the data of qRT-PCR, the expression of miR-424 was classified into low expression group (*n* = 69) and high expression (*n* = 27). **C, D & G, H.** Survival analysis of three subtypes of HCC. According to the clinical data, 96 HCC patients are divided into three subtypes (SHCC, SLHCC and NHCC). Survival curves were constructed using the Kaplan-Meier method and evaluated using the log-rank test.

To assess the association of miR-424 expression with HCC prognosis, analysis was conducted between two groups: one with relative high miR-424 expressions, while the other with relative low miR-424 expressions (cutoff value of ANLT/HCC more than two-fold). The results derived from two different cohorts (training cohort and validation cohort) were similar. miR-424 expression was significantly associated with tumor size (*P* < 0.001, *P* < 0.001), Tumor nodule number (*P* = 0.001, *P* = 0.045), TNM stage (*P* = 0.001, *P* = 0.019) and BCLC stage (*P* = 0.013, *P* = 0.008, Table 1 & Table S2), respectively. The data were analyzed with the Cox proportional hazards regression model. With univariate survival analysis, cirrhosis (*P* = 0.028, *P* = 0.036), tumor nodule number (*P* = 0.035, *P* = 0.032), vein invasion (*P* = 0.026, *P* = 0.012) and miR-424 expression (*P* = 0.013, *P* = 0.008) reached significance for overall survival (OS) and Disease-free survival (DFS), respectively. With multivariate survival analysis, we found that the OS and DFS of HCC patients was significantly dependent on tumor nodule number (*P* = 0.012, *P* = 0.025), vein invasion (*P* = 0.025, *P* = 0.016) and miR-424 expression levels (*P* = 0.032, *P* = 0.022; Table 2 & Table S3). Furthermore, HCC patients with high miR-424 expressions had much longer overall survival time (median survival time, 44.0 *vs*. 20.0 months, *P* = 0.007) than those with low miR-424 expressions (Fig. 2A). HCC patients with high miR-424 expressions also had longer disease-free survival time (median survival time, 36.0 *vs*.15.0 months, *P* = 0.003; Fig. 2B) than those with low miR-424 expression. In addition, the overall survival and disease-free survival time of three subtypes of HCC were also analyzed. Our data showed that SHCC and SLHCC patients had longer overall survival (37.0 *vs*. 14.0, *P* = 0.007 and 35.0 *vs*. 14.0 months, *P* = 0.008; Fig. 2C) and disease-free survival time than NHCC patients (34.0 *vs*. 13.0, *P* = 0.011 and 30.0 *vs*.13.0 months, *P* = 0.012; Fig. 2D). Of note, these results were further confirmed in the validation cohort, in which, we also found that HCC patients with high miR-424 expressions had much longer overall survival time (median survival time, 41.1 *vs*. 20.3 months, *P* = 0.009; Fig. 2E) and disease-free survival time (median survival time, 33.4 *vs*.14.6 months, *P* = 0.005; Fig. 2F) than those with low miR-424 expressions. The univariate and multivariate survival analysis in validation cohort yielded results similar to that of the training cohort (Table S4 & Table S5). Also, in validation cohort, SHCC and SLHCC patients also had longer overall survival (37.8.0 *vs*. 15.5, *P* = 0.001; 35.6 *vs*.15.5 months *P* = 0.004; Fig. 2G) and disease-free survival time than NHCC patients (35.2 *vs*. 14.5, *P* = 0.007; 32.4 *vs*.14.5 months, *P* = 0.010; Fig. 2H).

**Table 1:** Correlations between miR-424 expression level and clinicopathological variables of 96 cases of HCC in training cohort

Clinicopathologic Variables	*N*	miR-424 expression		*P* value
		Low	High	
Gender				
Male	80	60	20	
Female	16	9	7	0.139
Age(years)				
≤60	62	48	14	
>60	34	21	13	0.154
HBsAg				
Negative	29	22	7	
Positive	67	47	20	0.629
AFP				
Negative	11	5	6	
Positive	85	64	21	0.069
Cirrhosis				
Absence	36	24	12	
Presence	60	45	15	0.483
Child-Pugh Score				
A	31	20	11	
B	65	49	16	0.333
Tumor size (cm)				
≤5	28	10	18	
>5	68	59	9	**<0.001**
Capsular formation				
Presence	44	33	11	
Absence	52	36	16	0.650
Tumor nodule number				
Solitary	64	53	11	
Multiple(≥2)	32	16	16	**0.001**
TNM Stage				
I/II	76	61	15	
III	20	8	12	**0.001**
BCLC Stage				
0–A	66	53	13	
B–C	30	16	14	**0.013**
Edmondson-Steiner Stage				
I–II	33	22	11	
III–IV	63	47	16	0.476
Vein invasion				
Presence	17	10	7	
Absence	79	59	20	0.236

**Table 2:** The Cox regression analyses of overall survival (OS) and miR-424 expression level as well as clinicopathological parameters in training cohort

Variables	Univariable analysis		Multivariable analysis	
	HR (95% CI)	*P*	HR (95% CI)	*P*
Gender(Male *vs*. Female)	0.846 (0.333 − 2.148)	0.525		
Age(≤ 60 *vs.* > 60)	0.986 (0.447 − 2.175)	0.671		
HBsAg(Negative *vs*. Positive)	1.047 (0.617 − 2.113)	0.738		
Cirrhosis(Presence *vs*. Absence)	2.914 (1.126 − 7.543)	**0.028**	1.833 (0.702 – 4.053)	0.209
Child-Pugh Score(Grade A *vs*. B)	1.071 (0.747 − 2.221)	0.872		
AFP level(ng/ml, ≤ 20 *vs*. > 20)	0.762 (0.370 − 1.571)	0.262		
Tumor size(> 5 *vs*. ≤ 5 cm)	2.067 (0.861 − 2.964)	0.104	3.739 (0.961 – 4.758)	0.205
Capsular formation(Presence *vs*. Absence)	1.251 (0.483 − 3.457)	0.398		
Tumor nodule number(Solitary *vs*. Multiple)	3.147 (1.082 − 9.156)	**0.035**	4.728 (1.413 – 9.181)	**0.012**
Edmondson-Steiner Stage(I–II *vs*. III–IV)	1.101 (0.874 − 1.661)	0.597		
Vein invasion(Presence *vs*. Absence)	1.830 (1.238 − 3.643)	**0.026**	3.261 (1.158 – 9.181)	**0.025**
miR-424 expression(Low *vs*. High)	2.291 (1.250 − 6.827)	**0.013**	2.921 (1.096 – 7.787)	**0.032**

### miR-424 inhibits HCC growth *in Vitro* and *in Vivo*

**Figure 3:**
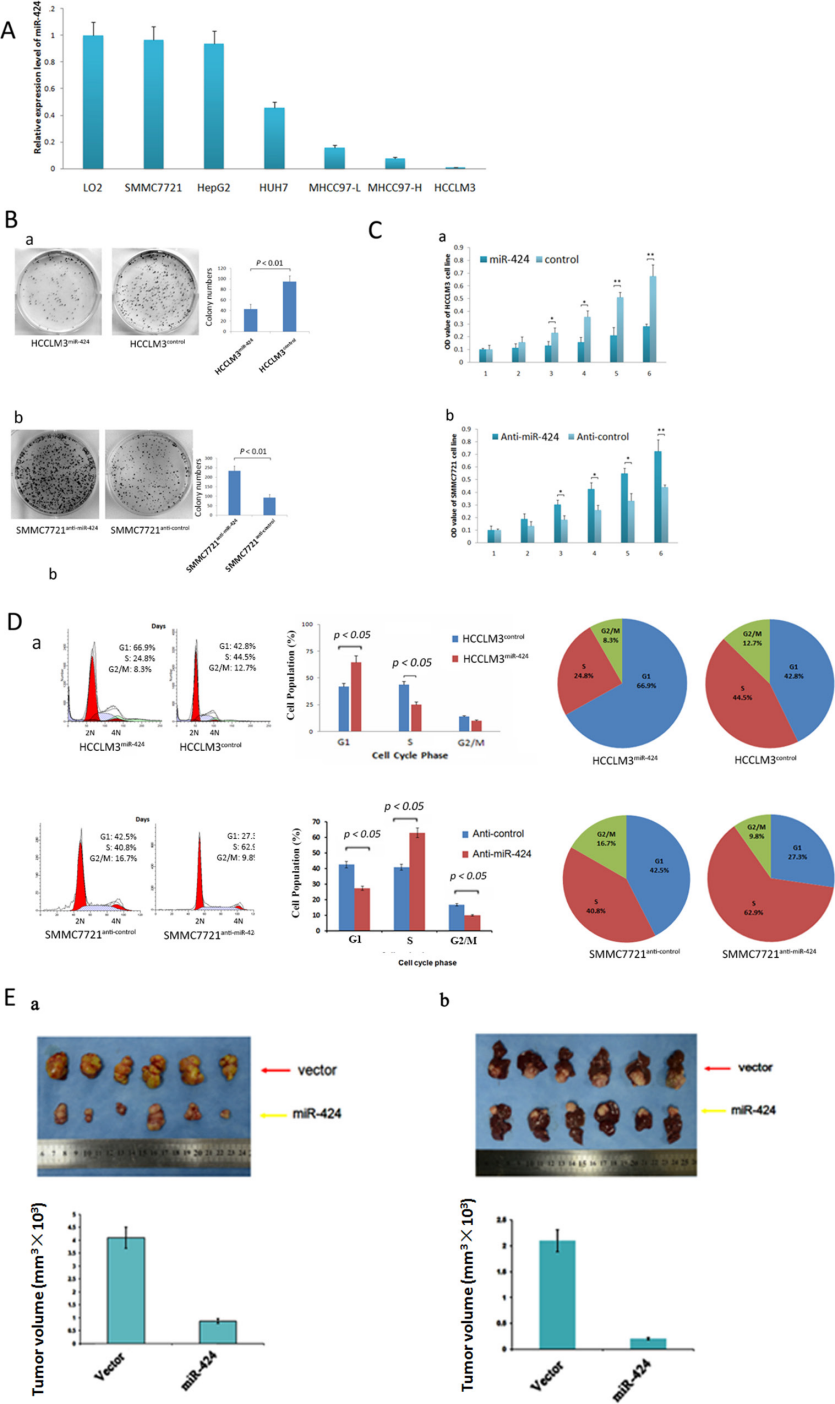
miR-424 inhibits HCC cell growth and colony formation *in vitro* and *in vivo* **A.** miR-424 expression in six liver cancer cell lines and normal liver cell line (L02). Expression levels of miR-424 were normalized to the corresponding levels of U6 snRNA. Each sample was analyzed in triplicate, and values are expressed as levels (mean ± standard deviation) relative to those in L02 cells. **B.** The colony formation assay was performed as described in Materials and Methods. The number of colonies was counted and compared. **C.** The growth of HCCLM3 and SMMC7721 cells with miR-424 overexpression or vector control was determined as described in Materials and Methods. *P < 0.01. **D.** The cell cycle distribution of HCCLM3 cells infected with miR-424 lentivirus or control vector were analyzed as described in Metrials and Methods. **E.** miR-424 inhibits HCC cell growth *in vivo*. The HCC mouse model in mice was constructed by using HCCLM3 cells infected with control vector or miR-424 lentivirus. The size of subcutaneous tumors and local liver tumors in these two groups was calculated and compared.

We measured miR-424 expression in six liver cancer cells (SMMC7721, HepG2, HUH7, MHCC97-L, MHCC97-H and HCCLM3), and normal liver cell line L02 were used as a control. The relative expression levels of miR-424 in these six HCC cells were 0.96, 0.93, 0.45, 0.15, 0.07and 0.04 as compared with that of L02 cells, respectively (Fig. 3A). Then, we constructed overexpressed miR-424 cell line (HCCLM3) and knockdown cell line (SMMC7721). The relative expression level of miR-424 was analyzed in these two cell lines by qRT-PCR (Fig S3). The expression level of miR-424 was significantly overexpressed in HCCLM3^miR-424^ cells and low expressed in SMMC7721^anti-miR-424^ cells (Fig S2).

To demonstrate the effect of miR-424 on HCC growth, we first performed colony formation assay *in vitro*. As shown in Fig. 3B, lentiviral induced ectopic miR-424 resulted in a significant decrease for cell colony formation in HCCLM3 cells. On the other hand, knock down of miR-424 in SMMC7721 cells by shRNA resulted in marked increase in cell colony formation. We also performed HCC cell proliferation assay in these two HCC cell lines. Ectopic miR-424 expression suppressed HCCLM3 cells proliferation and inhibition of miR-424 expression by shRNA accelerated the proliferation of SMMC7721 cells (Fig. 3C). In consistent with these results, cell cycle analysis revealed that miR-424 arrested the cells at G1/S checkpoint. Ectopic miR-424 expression increased the percentage of HCCLM3 cells in G1 phase but decreased the percentage of HCCLM3 cells in S phase. Inversely, depressed miR-424 expression decreased the percentage of SMMC7721 cells in G1 and G2 phase but increased the percentage of SMMC7721 cells in S phase (Fig. 3D). Besides, miR-424 also increased the apoptosis rate compared with controls in HCCLM3 cells, while anti-miR-424 decreased the apoptosis in SMMC7721 cells. (Fig S3). To confirm the above data *in vivo*, we created tumor xenograft mouse models. Consistently, miR-424 significantly inhibited tumor growth *in vivo*. The size of subcutaneous tumors and local liver tumors derived from miR-424 expressing SMMC7721 cells were dramatically smaller than that of vector expressing cells (*P* = 0.022, *P* = 0.013, respectively; Fig. 3E). Furthermore, our data also showed that overexpressed miR-424 changed HCC cells into a mesenchymal-like morphology, suggesting that miR-424 might alter the properties of EMT of HCC cells. Next, immunofluorescence analysis (IF), western blot and IHC analysis were performed in HCC cells and tissues. E-cadherin and vimentin were used as the epithelial and the mesenchymal marker, respectively. Western blot and IF analysis revealed that overexpression of miR-424 in HCCLM3 decreased the expression of vimentin and increased the expression of E-cadherin in HCC cells, whereas inhibition of miR-424 in SMMC7721 increased the expression of vimentin but decreased the expression of E-cadherin (Fig S4A & S4C). IHC analysis showed that the expression of E-cadherin was higher and vimentin was lower in miR-424 high expressed HCC tissues than those in miR-424 low expressed HCC tissues (Fig S4B). Taken together, our data supported a growth inhibitory activity of miR-424 in HCC *in vitro* and *in vivo*.

### Akt3 and E2F3 are candidate targets of miR-424

**Figure 4:**
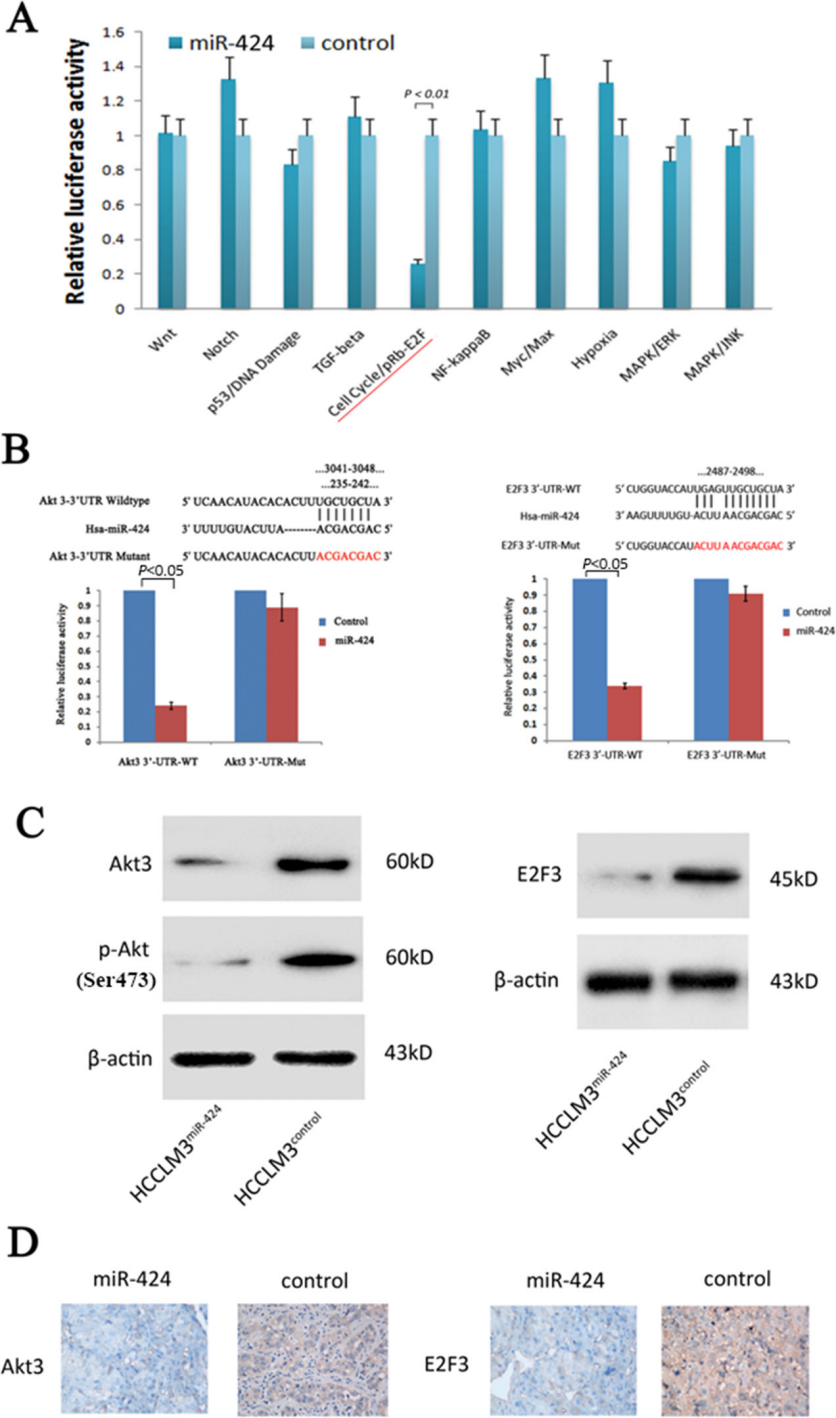
Akt3 and E2F3 are both direct downstream targets for miR-424 **A.** Multi-pathway reporter arrays were used as described in materials and methods for searching the possible signaling pathway of miR-424. **B.** The pGL3-Promoter (vector) of wild type and mutant type of Akt3 and E2F3 and pGL3-Promoter -miR-424 was constructed according miR-424 and its putative binding sequence in the 3′-UTR of Akt3 and E2F3 (3041–3048 and 235–242 for Akt3, 2487–2498 for E2F3). The mutant miR-424-binding site was generated in the complementary site for the seed region of miR-424. Relative luciferase activity was analyzed. HCCLM3 cells were cotransfected with pGL3-Promoter (vector) or pGL3-Promoter -miR-424, firefly luciferase reporter containing either a wildtype or a mutant 3′-UTR (indicated as WT or Mut on the X axis), and a Renilla luciferase expressing construct (as internal control to calibrate the differences in both transfection and harvest efficiencies). The firefly luciferase activity of each sample was normalized to the Renilla luciferase activity. The normalized luciferase activity of wildtype pGL3-transfectants in each experiment was set as relative luciferase activity. **C.** Western blot results of endogenous Akt3 and E2F3 proteins in HCCLM3 cells infected with miR-424 lentivirus or vector control. **D.** Analysis of Akt3 and E2F3 expressions in orthotopic imlplant primary tumors by IHC.

To understand the mechanisms by which miR-424 suppressed HCC growth, we searched for candidate targets of miR-424 that might play a role in HCC pathogenesis. We first used a multi-pathway reporter array to explore the potential signaling pathways of miR-424 regulated. As shown in Fig. 4A, miR-424 expression attenuated the activity of cell cycle/pRb-E2F signaling. Considering the crucial role of this pathway in the regulation of cell proliferation [20–22], we focused on it to search for the potential targets of miR-424. We used the miRanda, TargetScan and PicTar algorithms and only considered the targets detected by all three programs. We identified the 3′-UTR of Akt3 and E2F3 that were able to bind to the “seed region” of miR-424 (Fig. 4B). To confirm that miR-424 might be binded to the 3′-UTR of Akt3 and E2F3, we performed a luciferase assay using the constructs described in Fig. 4B. As predicted, miR-424 was bound to Akt3 and E2F3 3′-UTR, resulting in markedly reduced luciferase activities. This effect was specific as miR-424 failed to suppress the luciferase activity when the binding site within Akt3 and E2F3 3′-UTR was mutated (Fig. 4B). The results indicated that Akt3 and E2F3 are the downstream targets of miR-424. Moreover, Western blot analysis further demonstrated that ectopic miR-424 dramatically suppressed the endogenous protein levels of Akt3 and E2F3 in HCCLM3 cells (Fig. 4C). Besides, the expression level of Akt3 and E2F3 gradually decreased from patient samples with low miR-424 expression level to high expression level (Fig. S5). In addition, the expression levels of Akt3 and E2F3 in liver tumor tissues isolated from nude mice in Fig. 3E were determined by immunostaining. The expression levels of Akt3 and E2F3 in those liver tumor tissues with miR-424 overexpressed were down-regulated compared with control group (Fig. 4D).

### Both gain- and loss-of-function Studies Showed that Akt3 and E2F3 mediate the Role of miR-424 in regulation of HCC Cell Proliferation

**Figure 5:**
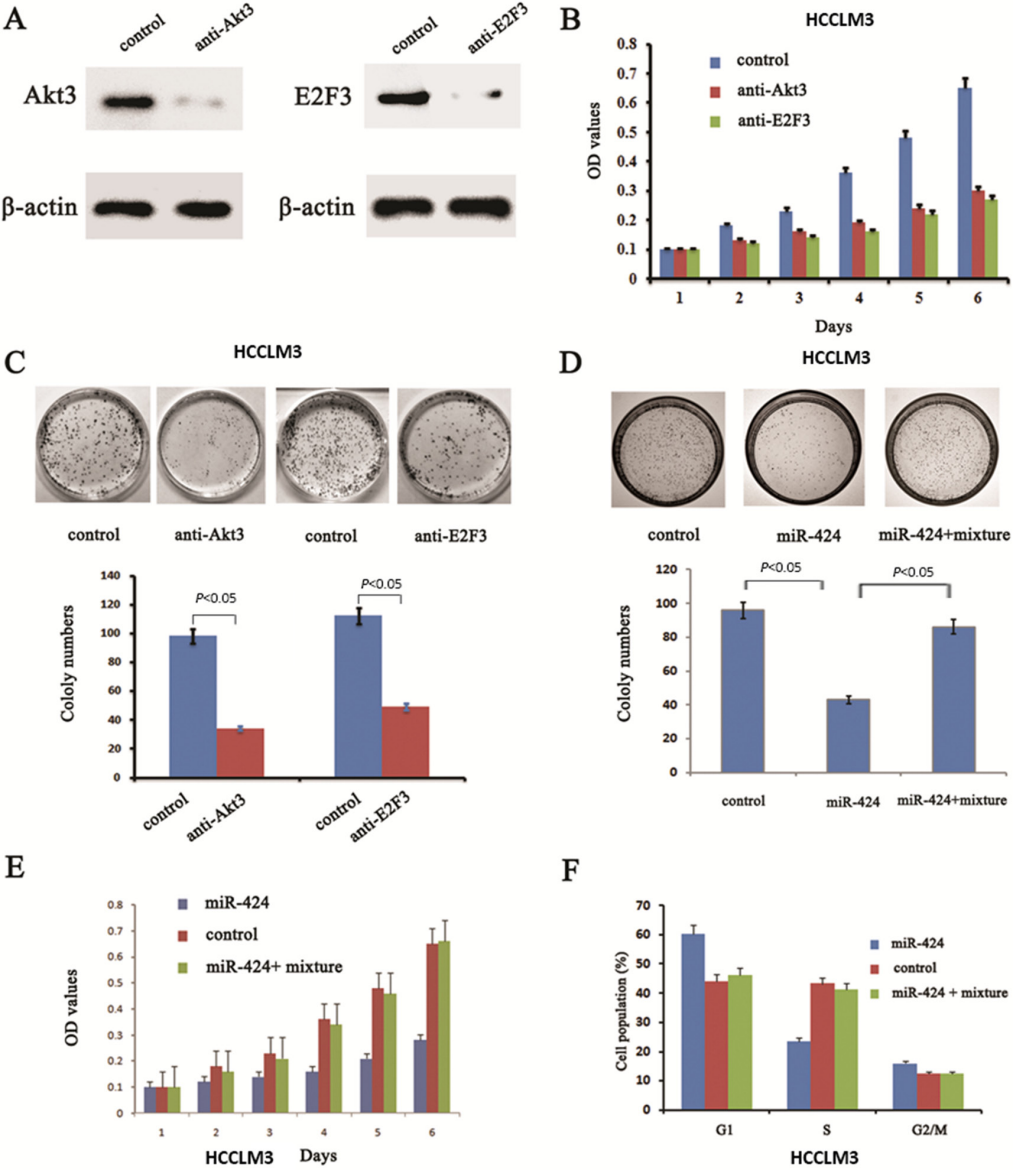
Blockage of Akt3 and E2F3 could mimic the effects of miR-424 expression in HCCLM3 cells and reintroduce both Akt3 and E2F3 into miR-424 transduced HCCLM3 cells abrogates the suppressive roles of miR-424 in HCC cell proliferation **A.** Western blot was performed to assess the inhibit efficiency of Akt3 and E2F3 siRNA. **B.** Cell proliferation analysis and **C.** colony formation assay were performed to examine the effect of Akt3 and E2F3 siRNA on the biological characteristics of HCC cells. **D.** colony formation assay, **E.** Cell proliferation analysis and cell cycle distribution assay **F.** were performed to validate reintroduce Akt3 and E2F3 into miR-424 transduced HCCLM3 cells abrogates the suppressive roles of miR-424 in HCC cell proliferation.

The above results prompted us to examine whether miR-424 suppresses HCC growth through repression of Akt3 and E2F3 expression. For this purpose, we first examined whether downregulation of Akt3 and E2F3 could mimic the effect of miR-424 expression. We introduced siRNA for Akt3 and E2F3 into HCCLM3 cells. Western blot analysis confirmed that the expression of Akt3 and E2F3 were inhibited (Fig. 5A). Indeed, both cell proliferation assay (Fig. 5B) and colony formation assay (Fig. 5C) confirmed that HCCLM3 cells transfected with Akt3 siRNA and E2F3 siRNA mimicked the effect of ectopic miR-424 expression on HCCLM3 cells. Conversely, both Akt3 and E2F3 were reintroduced into miR-424 expressing HCCLM3 cells to examine the effect on HCCLM3 cells proliferation. Our results showed that ectopic E2F3 and Akt3 expression in miR-424 expressing cells attenuated the inhibitory effect of miR-424 on HCC proliferation (Fig. 5D, 5E & 5F). Taken together, these results indicated that Akt3 and E2F3 were downstream targets of miR-424 in HCC cells.

### miR-424 Represses Cell Cycle/E2F Signaling by Direct Targeting Akt3 and E2F3 to Suppress HCC Growth and Survival

**Figure 6:**
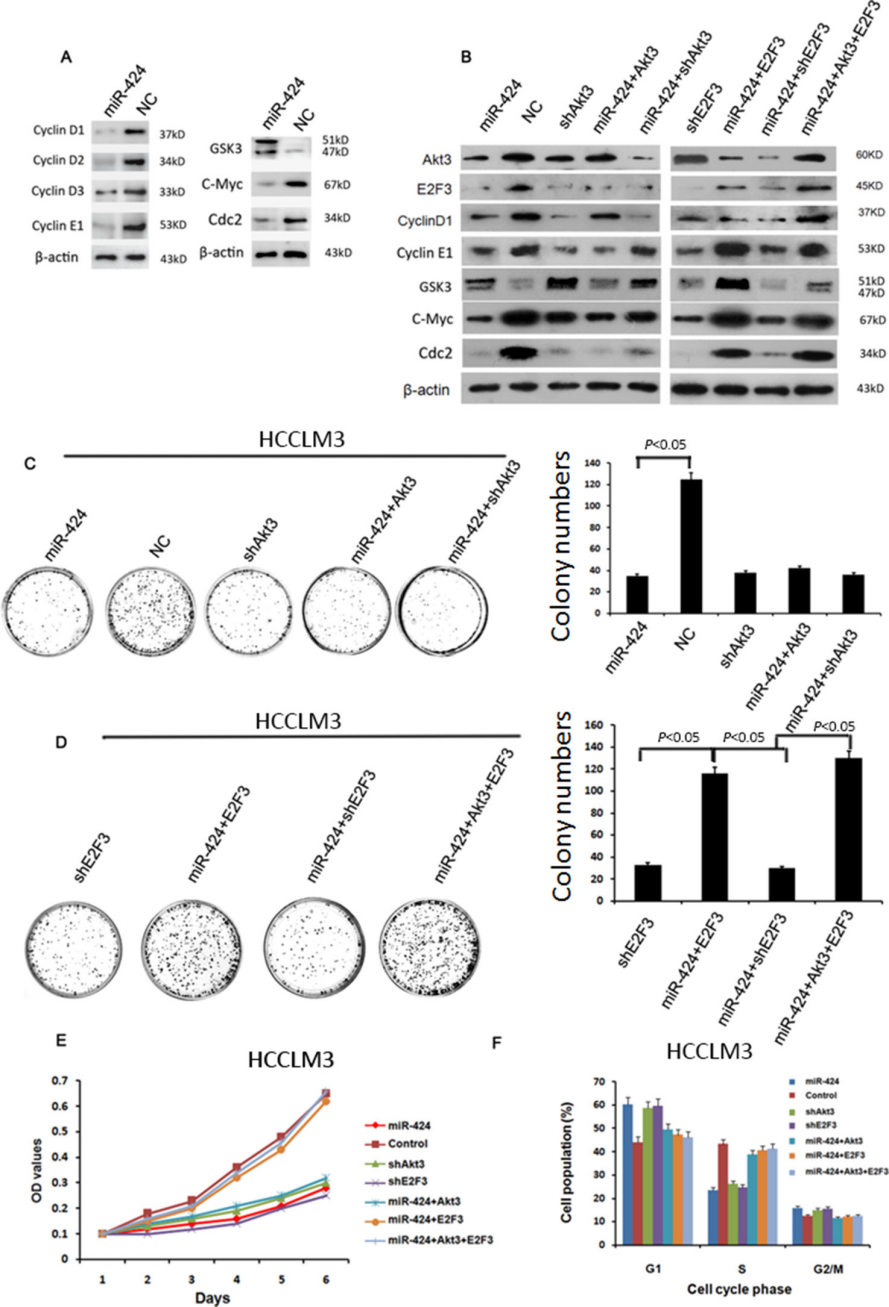
miR-424 represses the expression of cell cycle/E2F signaling **A.** overexpression of miR-424 inhibit the protein expression level of several members of Akt3/ E2F3 signaling. **B.** Western blot results showed the regulation relationships between miR-424 and its downstream proteins **C–F.** Colony formation assay (C&D), cell proliferation assay (E) and cell cycle analysis (F) showed that the inhibitory effect of miR-424 on HCC cell proliferation was abrogated by overexpression of mixture E2F3 and Akt3 or E2F3 alone, while this phenotype did not produced in miR-424 transduced cells only Akt3 overexpressed alone.

To further confirm the regulation of cell cycle/E2F by miR-424, western blot was performed to examine whether miR-424 regulated the protein expression of other members of cell cycle/E2F signaling besides Akt3 and E2F3. Interestingly, we found that some genes including Cyclin D1, Cyclin D2, Cyclin D3, Cyclin E1, GSK3, c-Myc, Cdc2 were also regulated by this microRNA as reflected at the protein levels in HCCLM3 cells (Fig. 6A), suggesting these genes were also in the downstream of miR-424. To substantiate the notion, we examined the expression of the above proteins in HCCLM3 cells expressing miR-424, vector (negative control), shAkt3, miR-424+Akt3, miR-424+shAkt3, shE2F3, miR-424+E2F3, miR-424+shE2F3, or miR-424+Akt3+E2F3, respectively. We found that Cyclin D and GSK3 were regulated by miR-424 and Akt3, while Cyclin E, C-Myc and Cdc-2 were regulated by miR-424 and E2F3 (Fig. 6B). In addition, Akt3 may be the upstream of E2F3. Altogether, the results suggest a regulatory network (Fig. S6). To examine the biological consequence of miR-424-mediatd regulation of cell cycle/E2F signaling network, we performed the colony formation assay (Fig. 6C & 6D), cell proliferation assay (Fig. 6E) and cell cycle analysis (Figure 6F) in HCCLM3 cells, MHCC97-L (Fig. S7A–S7C) and MHCC97-H cells (Fig. S7D–S7F). The results were consistent with a role for miR-424 targeting Akt3 and E2F3 in suppression of HCC growth. In addition, these results also implicate that Akt3 is the upstream of E2F3 in miR-424/Akt3/E2F3 axis. Taken together, our data support the model in which miR-424 represses cell cycle/E2F pathway by targeting Akt3 and E2F3 resulting in suppressing HCC growth and improving patients' survival after liver resection.

## DISCUSSION

HCC is a primary neoplasm of the liver and the fourth most common cause of death from cancer worldwide [1, 23]. However, its underlying molecular mechanism remains largely unknown. It is well known that each subtype of HCCs exhibits a distinct clinicopathological and molecular characteristics [4, 5]. Previously, we defined a specific subtype of HCC named as SLHCC [4, 9]. Interestingly, although SLHCC is larger in size, it showed similar clinical outcomes as SHCC after liver resection. Both of them are better than NHCC in terms of outcomes. Our findings do not support the concept that HCC in large size has reached an advanced stage and cannot be resected. According to this finding, many patients with SLHCC have been cured [4]. Therefore, clarification of the molecular pathogenesis of HCC, especially SLHCC is crucial for developing effective intervention and therapeutic strategies to improve the outcome of patients with this devastating disease.

Altered expression of miRNAs has been reported to contribute to the initiation and progression of cancer [24–26]. Studies have shown that more than 50% of miRNAs are located in cancer associated genomic regions or in fragile sites [12]. Oneyama et al had demonstrated that the tumorigenicity and invasive activity of human prostate and colon cancer cells were suppressed by miR-424 [27]. Moreover, previous studies had reported that miR-424 induced cell cycle arrested by down-regulating Cdc25A in mammary epithelial cells and muscle differentiation [28–29]. Meanwhile, Lei Yu et al found that miR-424 is down-regulated in hepatocellular carcinoma and suppresses cell migration and invasion through c-Myb [30]. However, the mechanism of its impact on HCC cell growth and survival is still unclear. Moreover, whether miR-424 could be a novel prognostic marker for HCC patients need to be clarified. In the present study, we performed a miRNA microarray to screen miRNAs relevant to HCC pathogenesis. We found that miR-424 was frequently down-regulated in both HCC tissues and liver cancer cell lines. Moreover, its expression level in SLHCC was close to SHCC but much lower in NHCC. Interestingly, miR-424 expression was significantly correlated with tumor size, tumor nodule number, TNM stage, vein invasion, overall survival and disease-free survival of HCC. Given that miR-424 was down-regulated in HCC tissues and liver cancer cell lines, we hypothesized that up-regulation of miR-424 might suppress the malignant phenotypes of HCC cells. The results derived from *in vitro* cell proliferation, colony formation, cell cycle analysis and *in vivo* tumor formation confirmed that ectopic miR-424 expression suppressed HCC cell proliferation. Recently, Drasin et al have shown that miR-424 plays distinct roles in tumor progression, potentially facilitating earlier, but repressing later stages of metastasis by regulating an EMT-MET axis [31]. Our supplementary data also showed that inhibition of miR-424 changed HCC cells into a mesenchymal-like morphology. Altogether, both clinical and experimental data supported a tumor suppressive role of miR-424 in HCC. The close correlation of miR-424 expression and HCC patient survival suggested that miR-424 could potentially be used as a biomarker to clinically predict the prognosis for patients with hepatocellular carcinoma.

The fundamental function of miRNAs is to regulate their target genes by direct cleavage of the mRNA and/or by inhibition of protein synthesis, according to the degree of complementarity with the target mRNA 3′UTR [32]. Our studies revealed that miR-424 suppressed the activity of cell cycle/pRb-E2F signaling pathway. Computational algorithms allowed us to predict the putative targets of miR-424 and help to identify some members of cell cycle/pRb-E2F signaling pathway such as Akt3 and E2F3 containing a putative miR-424 binding site within their 3′UTR region. This prediction was validated by luciferase activity assay. In addition, western blot analysis showed that the protein expressions of Akt3 and E2F3 were post-transcriptionally down-regulated by overexpression of miR-424. The importance of Akt3 and E2F3 in mediating the effect of miR-424 was substantiated by the finding that down regulation of miR-424-induced HCC proliferation was abolished by reintroduction of Akt3 and E2F3. In addition, the protein expressions of some other members of cell cycle/ E2F signalling such as cyclin D, cyclin E, GSK3, c-Myc and cdc2 were also down-regulated by overexpression of miR-424. Our data together uncovered miR-424 as a new member to the cell cycle/ E2F pathway and showed the miR-424/Akt3/E2F3 axis function as a suppressor in HCC growth and survival.

In conclusion, miR-424 is down-regulated in HCC and functions as a tumor suppressor suppressing HCC growth via targeting cell cycle/ E2F signalling. The identification of MicroRNA-424/Akt3/E2F3 axis in HCC would help to better understand the molecular mechanisms underlying HCC development. All of these would carry important implications in HCC intervetion/prevention and treatment.

## MATERIALS AND METHODS

### Patients and tissue specimens

Matched fresh HCC specimens and adjacent nontumorous liver tissues (ANLTs) were obtained from 96 patients during hepatic resection at the Department of Surgery, Xiangya Hospital of Central South University (CSU) from January 2002 to October 2006. These patients were divided into 3 subtypes :SLHCC, nodular HCC (NHCC, node number ≥ 2) and small HCC (SHCC, tumor ≤ 5 cm), and every subtype had 32 patients. We defined this cohort as training cohort. At the end of the first part of the study, 70 cases of matched fresh HCC specimens and ANLTs from a second set of patients were similarly collected from November 2006 to March 2010 to serve as the validation cohort. All research protocols strictly complied with REMARK guidelines for reporting prognostic bio-markers in cancer [33]. Diagrammatic sketch of patients enrolled and groups designed were showed in Fig S1. Details of patients in these two cohorts were described in the Table S1. Prior informed consent was obtained and the study protocol was approved by the Ethics Committee of Xiangya Hospital of CSU.

### miRNA array

miRCURY LNA™ microRNA chips (version 8.0, Exiqon, Vedbaek, Denmark) were used to profile the differences for miRNA expressions among SLHCC, SHCC and NHCC. The array contained a total of 840 specific probes in triplicates. It was performed according to the protocol of miRCURY LNA microRNA Array Power Labeling kit (Exiqon) [34]. The image analysis was conducted in Genepix Pro 6.0 (Axon Instruments) as described before [35] (details were described in the Supplementary Materials and Methods).

### Cell lines and cell culture

Six HCC cell lines and normal liver cell line L02 were used for this study. Details were described in the Supplementary Materials and Methods.

### Quantitative real-time PCR (qRT-PCR)

qRT-PCR was performed using TaqMan^®^ MicroRNA reverse transcription kit and TaqMan^®^ Universal PCR Master Mix (Ambion, TX) and SYBR Green Real-time PCR Master Mix (Toyobo, Osaka, Japan). Details were described in the Supplementary Materials and Methods.

### Western blot analysis

Details were described in the Supplementary Materials and Methods.

### Follow-up and prognostic study

Follow-up data were obtained after hepatic resection for all 166 patients. Details were described in the Supplementary Materials.

### Vector construction and transfection

DNA fragment for miR-424 was amplified from genomic DNA and inserted into Age I/EcoR I site of a lentiviral expression vector pGCSIL-GFP (GeneChem, Shanghai, China). The Akt3 and E2F3 expression vectors were constructed by inserting their ORF sequence into the pGCL vector (GeneChem, Shanghai, China). siRNAs were purchased from GenePharma company (Shanghai, China). Transfection was performed according to the manufacture's protocol. Viruses were harvested 72 hours after transfection and viral titers were 1 × 10^9^ TU/ml. 1 × 10^5^ cells were infected with 2 × 10^6^ lentivirus in the presence of 6 ug/ml polybrene (Sigma, MO). In the present study, the infection efficiency of lentivirus was over 90% (Figure S2). No significant cell death was observed after virus infection. Bulk transfectants were used for subsequent assays. For luciferase analysis, the 3′-UTR sequence of Akt3 and E2F3 were amplified from human liver genomic DNA and then cloned into the downstream region of a firefly luciferase cassette in the pGL3-Promoter vector (Promaga, Madison, WI) as instructed.

### Colony formation assays, cell proliferation and cell cycle analysis

Details were described in the Supplementary Materials and Methods.

### Multi-pathway reporter array

A Cignal Finder 10-Pathway Reporter Array (QIAGEN, MA, USA) was employed for the study. Reverse transfection technique was implemented. Cells were treated with overexpression miR-424 or negative control. Relative firefly luciferase activity was calculated and normalized to the constitutively expressed Renilla luciferase.

### Luciferase reporter assay

Luciferase activity was assessed according to the Dual-Luciferase Reporter Assay protocol (Promega, Madison, WI) using a Veritas™ 96-well Microplate Luminometer (Promega, Madison, WI) with substrate dispenser (Promega, Madison, WI). HEK293T cells transduced with leti-miR-424 or control virus were seeded in 96-well plates with 70% confluence. 12 hours later, the cells were cotransfected with 50 ng pGL3-Promoter -UTR and 10 ng pRLTK using the Lipofectamine LTX. After 24 hours of transfection, the cells were harvested for firefly and Renilla luciferase activity assay. The renilla luciferase activities were used to normalize the transfection efficiency.

### HCC mouse model

The hepatocellular carcinoma model in nude mice was constructed as described before [8]. Details were described in the Supplementary Materials and Methods. All animal studies were conducted in the Animal Institute of CSU according to the protocols approved by the Medical Experimental Animal Care Commission of CSU.

### Immunohistochemistry

The expression levels for ki-67, Akt3, E2F3, E-cadherin, Vimentin, PCNA and HIF-1 alpha in the local tumor tissues and HCC tissues were determined by immunostaining with antibodies against ki-67, Akt3 E2F3, E-cadherin, Vimentin, PCNA and HIF-1 alpha, respectively (Santa Cruz, CA).

### Statistical analysis

Statistical analysis was performed using the SPSS (version 16.0, Chicago, IL). Data for miR-424 expression in fresh specimens were analyzed by the Mann–Whitney *U*-test. Fisher's exact test was used for statistical analysis of categorical data. Spearman correlation test was used for analyzing the correlations between miR-424 expression level and the clinical and pathological variables. Survival curves were constructed by using the Kaplan-Meier method and evaluated by using the log-rank test. The cox proportional hazard regression model was used to identify factors that were independently associated with overall survival. In any case, *P* < 0.05 was considered with statistical significance.

## SUPPLEMENTARY DATA FIGURES AND TABLES



## Abbreviations

HCC: hepatocellular carcinoma
SLHCC: solitary large hepatocellular carcinoma
NHCC: nodular hepatocellular carcinoma
SHCC: small hepatocellular carcinoma
qRT-PCR: Real-time quantitative PCR
