# Supplementary material for: MicroRNA-424 inhibits Akt3/E2F3 axis and tumor growth in hepatocellular carcinoma
Source: Oncotarget. 2015 Aug 3;6(29):27736–50. doi: 10.18632/oncotarget.4811 (PMC4695022; doi:10.18632/oncotarget.4811)
Supplement: Supplementary file 1 [file oncotarget-06-27736-s001.pdf]

SUPPLEMENTARY DATA

Patients and tissue specimens

From January 2002 to March 2010, a total of 486 pairs of HCC and ANLTs were gathered from patients performed liver resection at Department of Surgery, Xiangya Hospital of Central South University. The training cohort contained randomly selected 96 cases in 210 patients from January 2002 to October 2006. The validation cohort contained randomly selected 70 cases in 208 patients from November 2006 to March 2010 (Supplementary Figure 1). The samples were snap-frozen in liquid nitrogen and stored at −80°C for later RNA extraction or formalin-fixed and paraffin embedded for imunohistochemistry. Histopathology was evaluated by two certified pathologists in the Department of Pathology at Xiangya Hospital of Central South University. The clinical and pathological features of these patients were described in Supplementary table 1. All research protocols strictly complied with REMARK guidelines for reporting prognostic biomarkers in cancer [1]. All human materials were obtained with informed consent and approved by the Ethics Committee of Xiangya Hospital of Central South University.

miRNA array

miRCURY LNA™ microRNA chips (version 8.0, Exiqon, Vedbaek, Denmark) were used to profile the differences for miRNA expression among SLHCC, SHCC and NHCC. The array contained a total of 840 specific probes in triplicates. Total RNA was extracted using mirVana™ miRNA isolation kit (Ambion, TX) from each selected sample, and equal amount of RNA from each subtype of HCC (SLHCC, SHCC and NHCC) was next mixed as a pool, respectively. The pooled RNA mixtures were 3′-end-labeled according to the protocol of miRCURY LNA microRNA Array Power Labeling kit (Exiqon). Hybridization of labeled RNA to the array was performed on a Tecan HS Pro 4800 (Tecan Group Ltd. Männedorf Switzerland) hybridization station. Slides were scanned using the Genepix 4000B (Axon Instruments, CA) microarray scanner, and image analysis was conducted in Genepix Pro 6.0 (Axon Instruments) as described before [2].

Cell lines and cell culture

HCCLM3 and SMMC7721 cell lines were kindly provided by the Liver Cancer Institute of Fudan University, Shanghai, China. HepG2 cell line was purchased from the American Type Culture Collection (ATCC, MA, USA). L02 cells were obtained from the Tumor Institute of Central South University, Changsha, China. These cells were cultured in High glucose Dulbecco's modified Eagle media (GIBCO BRL, Gaithersburg, MD) supplemented with 10% fetal bovine serum (HyClone, Logan, UT) and 5% CO2 at 37°C.

Quantitative real-time PCR (qRT-PCR)

qRT-PCR was performed using TaqMan® MicroRNA reverse transcription kit and TaqMan® Universal PCR Master Mix (Ambion, TX). For analysis of miR-424 and RNU6B expression (U6 snRNA, a reference gene), we employed a two-step qRT-PCR with specific primers for miR-424 and RNU6B (designed by Applied Biosystems) following the manufacturer's protocol. Real time RT-PCR was performed using a PRISM 7300 Sequence Detection System (Applied Biosystems, CA), in which each reaction (25 ul) contained 10 ul PCR Master Mix (Ambion, TX,) and 1.33 ul RT product, and each sample was analyzed in triplicates. PCR was carried out at 95°C for 10 min, followed by 40 cycles of amplification at 95°Cfor 15 s and 60°C for 60 s. Results are representative of two independent assays. Relative fold changes of expression in tumor tissues against ANLTs samples and among different cell lines were calculated using the comparative Ct ($2^{-\Delta\Delta Ct}$) method with U6 small nuclear RNA (Ambion, TX) as the endogenous control.

Western blot analysis

Total proteins were extracted and separated by sodium dodecyl sulfate-polyacrylamide gel electrophoresis (SDS-PAGE) and then transferred onto PVDF membrane (Millipore, Bedford, MA). The blotted membranes were incubated with antihuman Akt3 or E2F3 antibody (1:1000, Santa Cruz Biotechnology, Santa Cruz, CA), and then probed with a secondary antibody (1:3000, Santa Cruz Biotechnology). Beta-actin was used as a loading control.

Cell proliferation, cell cycle analysis and colony formation assays

Cell proliferation was determined by counting the number of cells using TC10™ automated cell counter (Bio Rad, CA). Cell cycle analysis was conducted by flow cytometry using a Propidium Iodide (PI) cell cycle detection kit (Beyotime Institute of Biotechnology, Beijing, China). For colony formation assays, 500 cells were seeded into 35mm dishes (Corning, NY) and cultured for 2 weeks at 37°C. The numbers of colonies per dish were counted after staining with crystal violet. All studies were conducted with 3 replicates.

HCC mouse model

The hepatocellular carcinoma model in nude mice was constructed as described before [3]. Briefly, 5×10^6 SMMC7721 cells were injected subcutaneously into the left upper flank regions of nude mouse (3–4 weeks of age, male, BALB/c). The subcutaneous tumor tissues were removed one month later and then implanted into the liver of nude mouse (5 in each group). After 6 wk of implantation, the mice were sacrificed, and the size for tumors was calculated as follows: tumor volume (mm3) = $(L \times W^2)/2$, where L = long axis and W = short axis. [4] Livers were collected and fixed with phosphate-buffered neutral formalin. Serial sections were subjected to histological analysis by hematoxylin and eosin (H&E) staining. The expression levels for Akt3 and E2F3 in the local tumor tissues were determined by immunostaining with antibodies against Akt3 and E2F3 (Santa Cruz, CA). All animal studies were conducted in the Animal Institute of CSU according to the protocols approved by the Medical Experimental Animal Care Commission of CSU.

REFERENCES

1. McShane LM, Altman DG, Sauerbrei W, Taube SE, Gion M, Clark GM, et al. Reporting recommendations for tumor marker prognostic studies (REMARK). J Natl Cancer Inst. 2005; 97:1180–1184.
2. Xia L, Huang W, Tian D, Zhu H, Qi X, Chen Z, et al. Overexpression of forkhead box C1 promotes tumor metastasis and indicates poor prognosis in hepatocellular carcinoma. Hepatology. 2013; 57:610–624.
3. Wu F, Yang LY, Li YF, Ou DP, Chen DP, Fan C. Novel role for epidermal growth factor-like domain 7 in metastasis of human hepatocellular carcinoma. Hepatology. 2009; 50:1839–50.
4. Zhang JF, He ML, Fu WM, Wang H, Chen LZ, Zhu X, et al. Primate-specific microRNA-637 inhibits tumorigenesis in hepatocellular carcinoma by disrupting signal transducer and activator of transcription 3 signaling. Hepatology. 2011; 54:2137–2148.

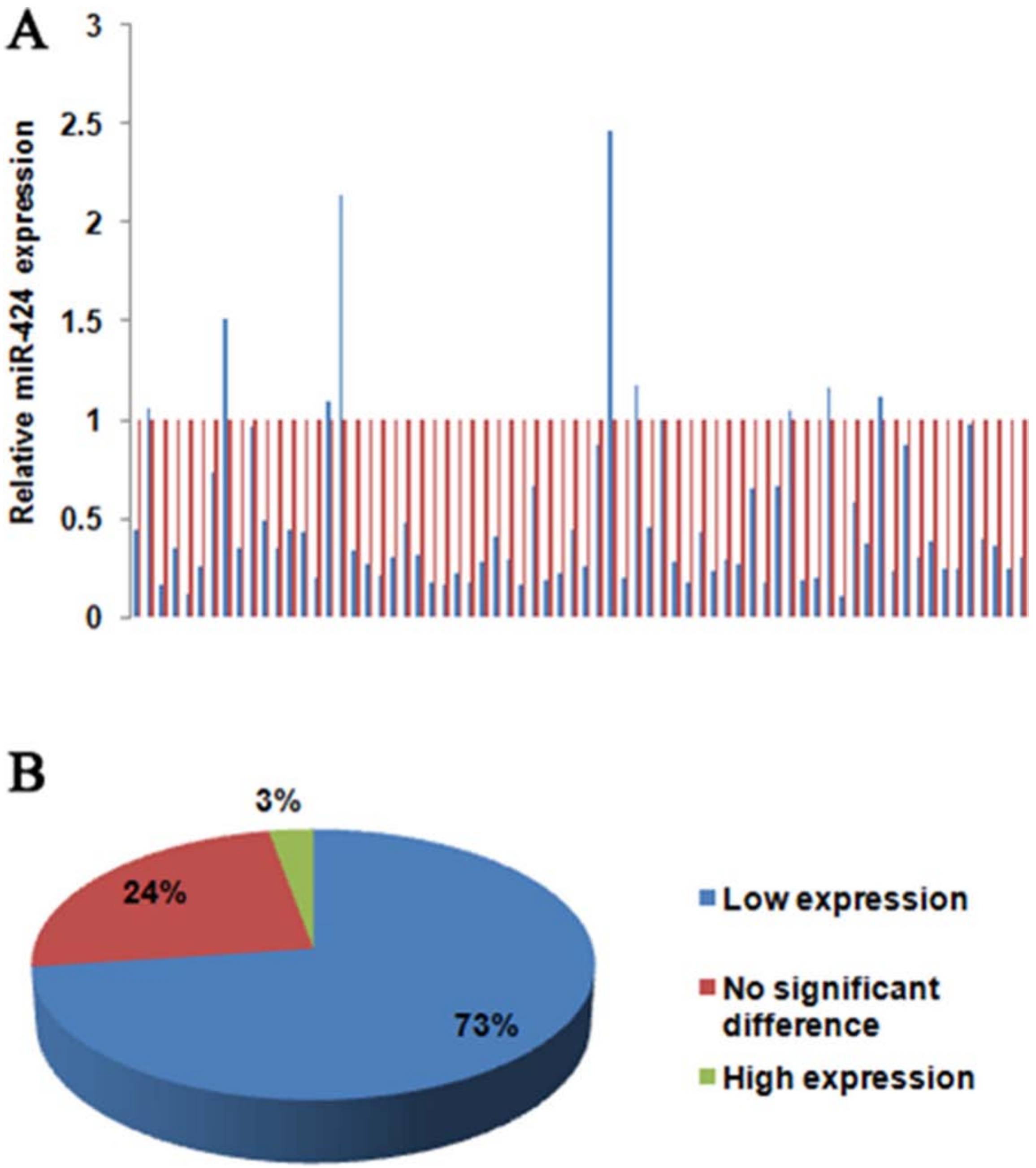

Supplementary Figure S1: Expression of miR-424 was also often down-regulated in validation cohort. Expression of miR-424 in 70 pairs of HCC tissues and the corresponding ANLTs. Expression levels of miR-424 were normalized to the corresponding levels of U6 snRNA.

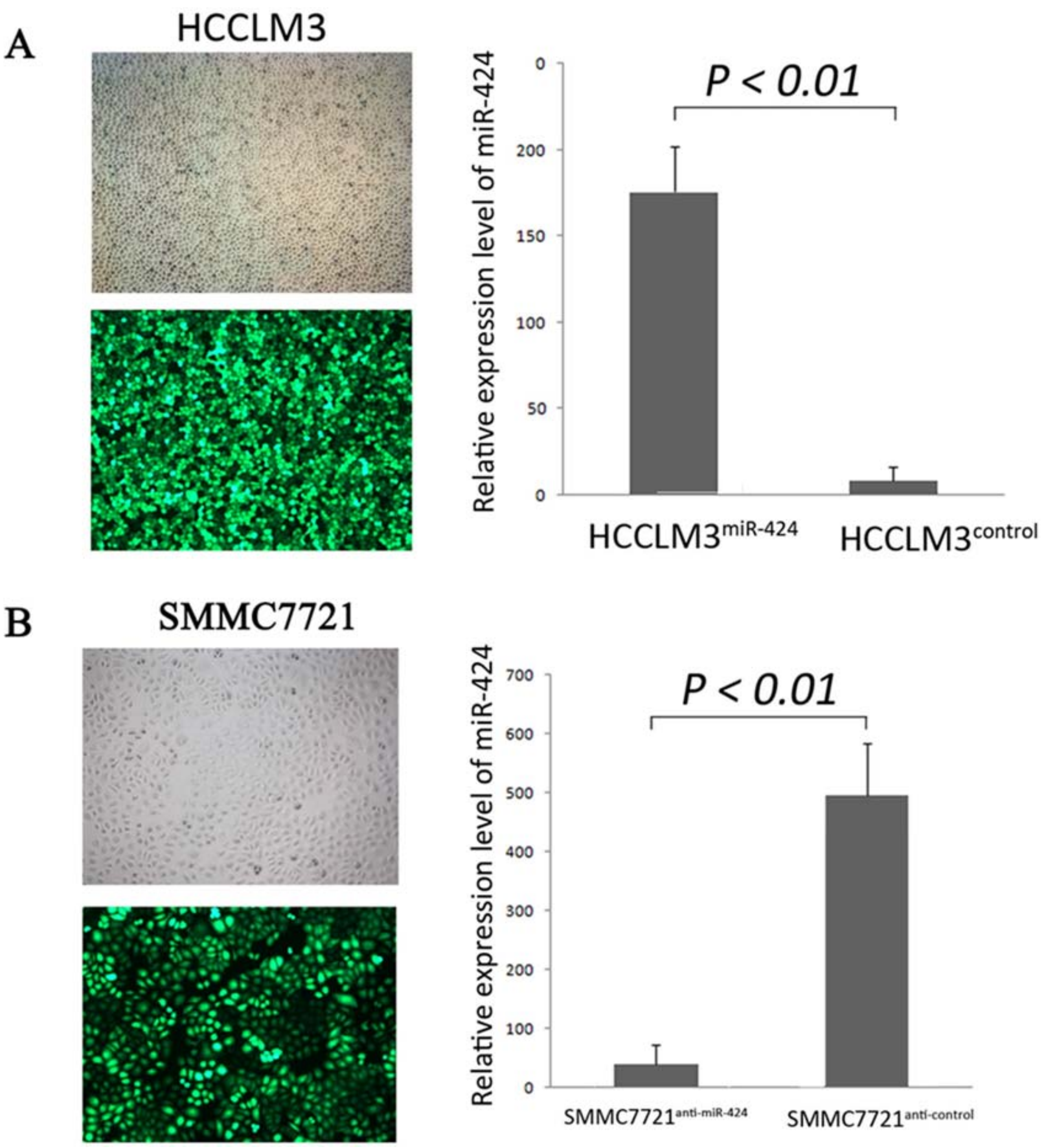

Supplementary Figure S2: The infection efficiency of lentivirus in HCCLM3. A. and SMMC7721 cells **B.** Due to this vector contains a GFP fragment, the cells will emits green fluorescence if they are infected by virus in green fluorescence sight. HCC cells are observed in light field and green fluorescence sight, respectively. The infection efficiency of virus was over 90% and there was no difference between miR-424 group and vector group. The overexpressed and inhibtion efficiency of these lentivirus were also examined.

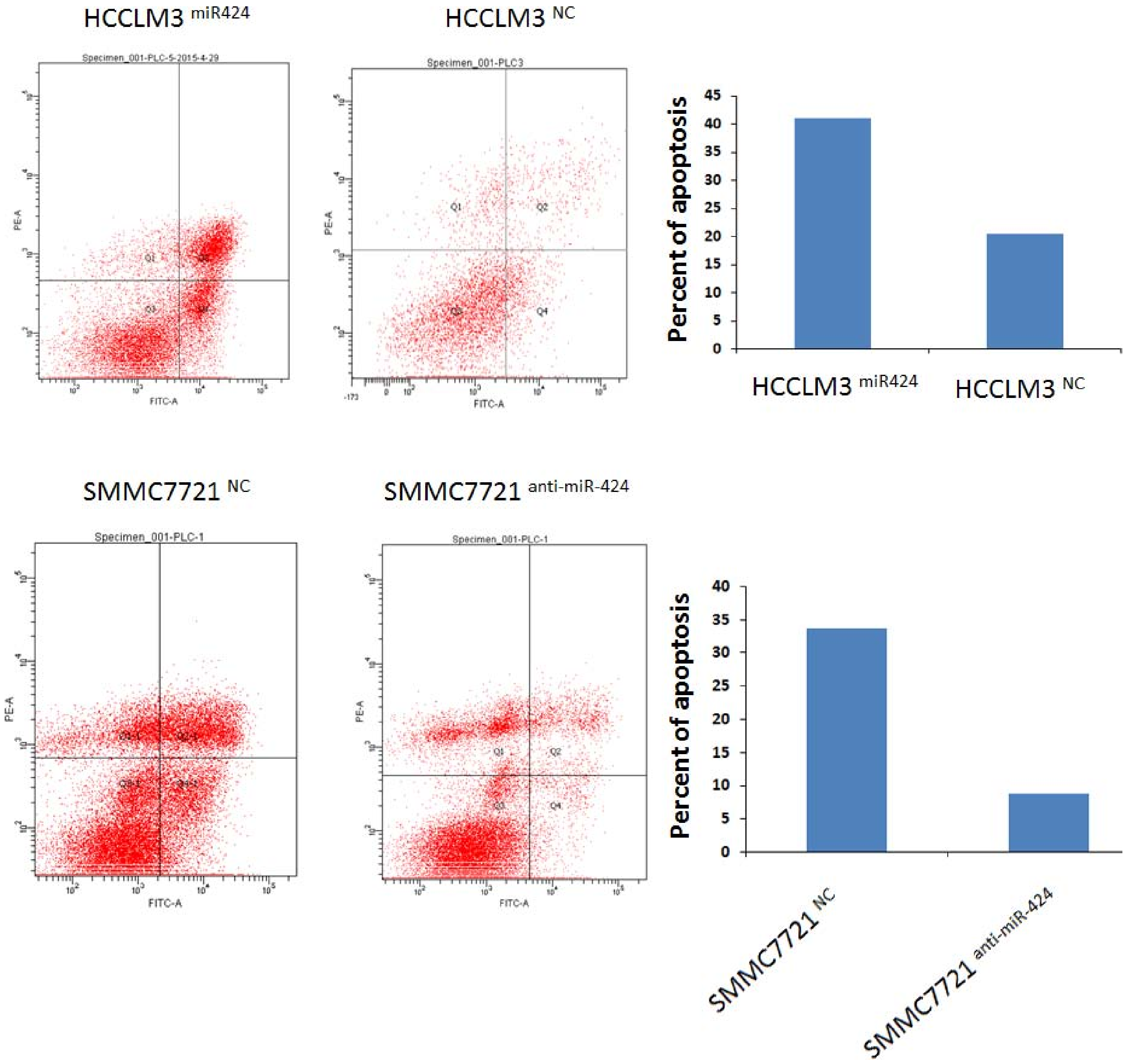

Supplementary Figure S3: The effect of miR-424 on HCC cell apoptosis. miR-424 increased the apoptosis rate compared with controls in HCCLM3 cells, while anti-miR-424 decreased the apoptosis in SMMC7721 cells.

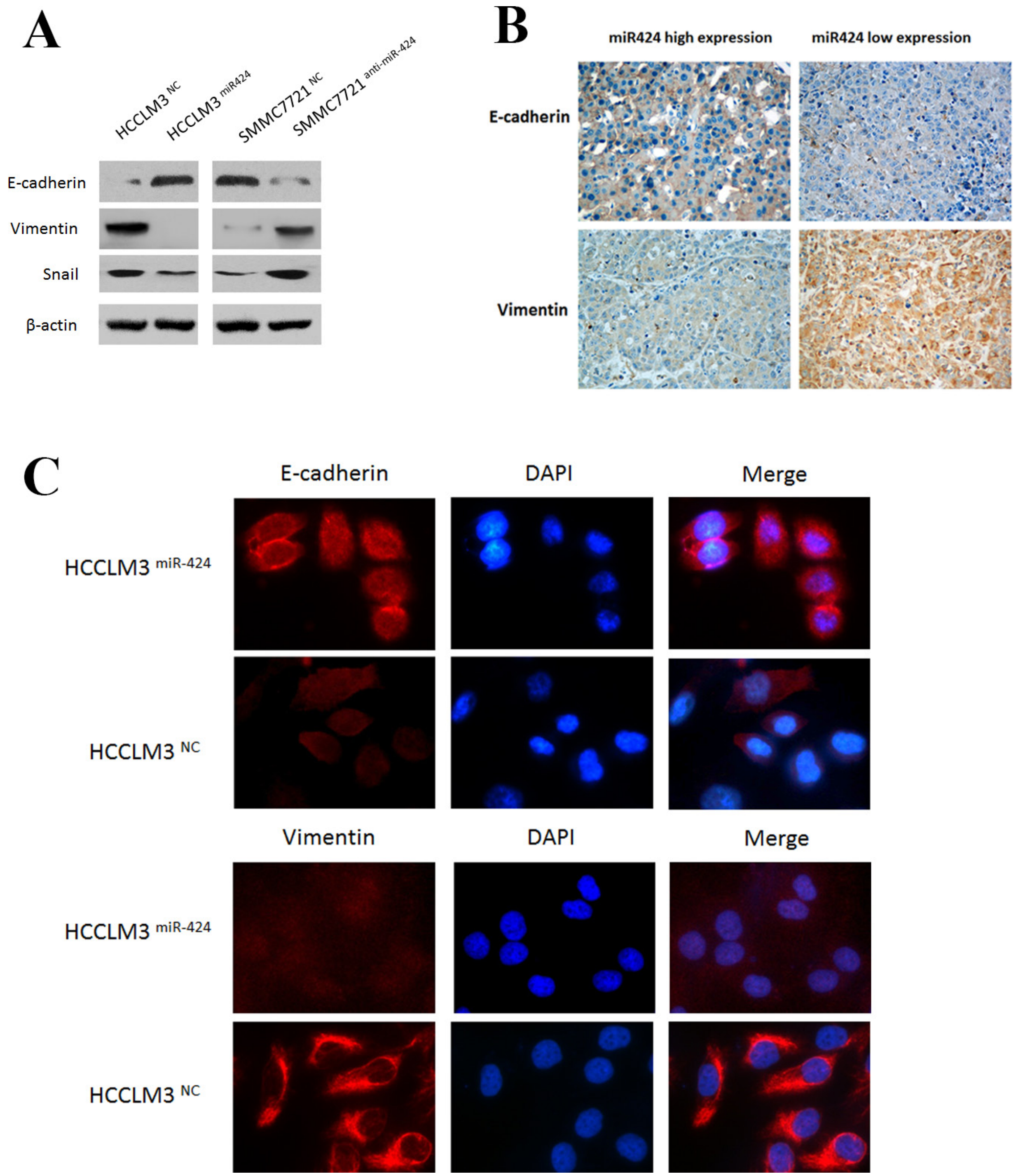

Supplementary Figure S4: miR-424 inhibited EMT in HCC cells and tissues. A. The WB for E-cadherin, Vimentin and Snail were performed in HCCLM3$^{miR\text{-}424}$, HCCLM3^{NC}, SMMC7721$^{anti\text{-}miR\text{-}424}$ and SMMC7721^{NC} cells. **B.** IHC for E-cadherin and Vimentin were performed in miR-424 high expressed HCC tissues and miR-424 low expressed HCC tissues. **C.** IF for E-cadherin and Vimentin were performed in HCCLM3$^{miR\text{-}424}$, HCCLM3^{NC}, SMMC7721$^{anti\text{-}miR\text{-}424}$ and SMMC7721^{NC} cells.

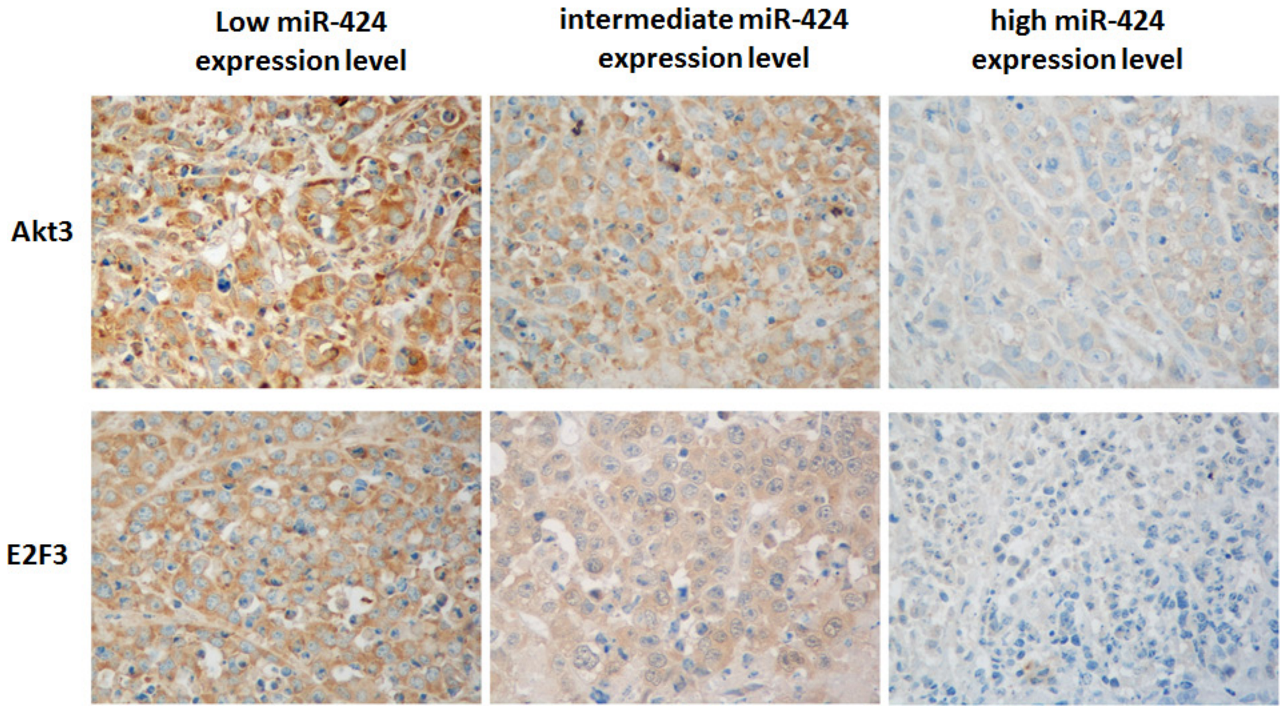

Supplementary Figure S5: IHC for Akt3 and E2F3 were performed in high miR-424 expression level, intermediate miR-424 expression level and low miR-424 expression level HCC tissues.

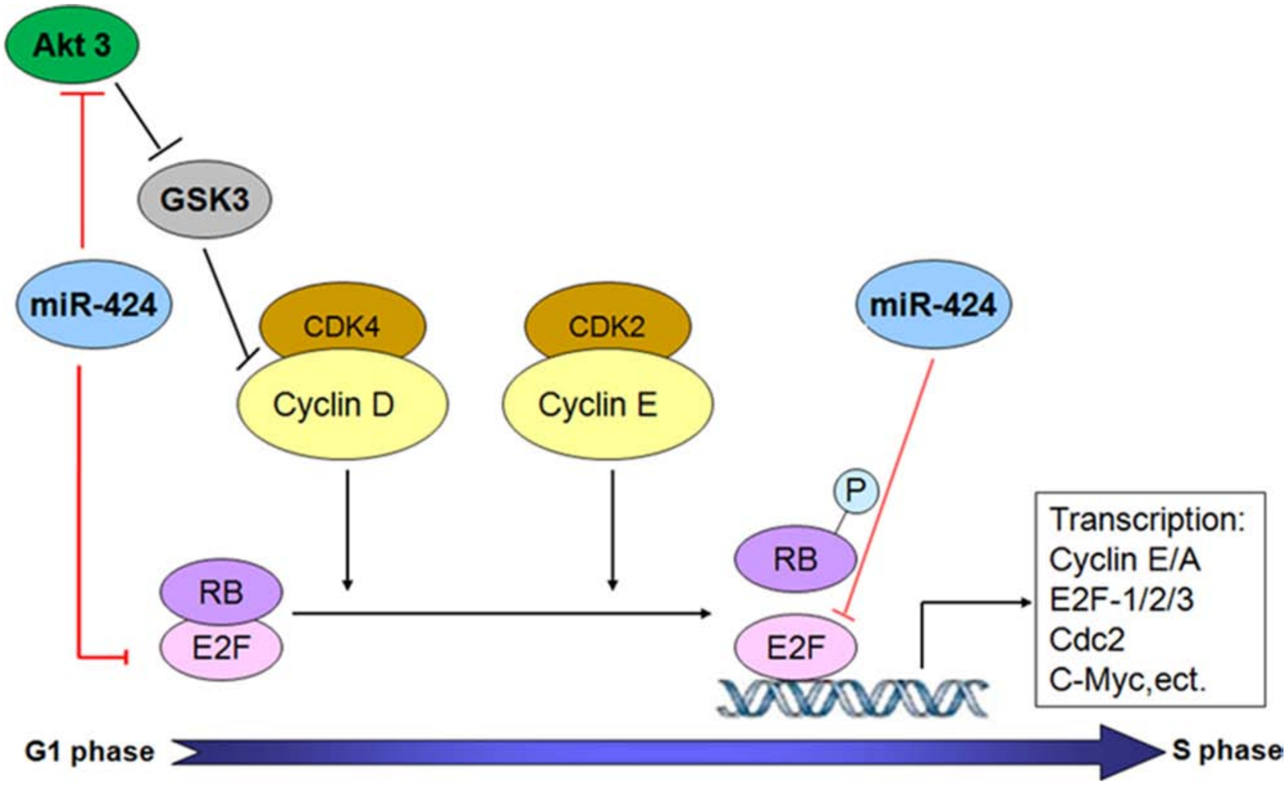

Supplementary Figure S5: Schematic representation of the major molecular mechanism of miR-424 suppresses tumor proliferation and metastasis.

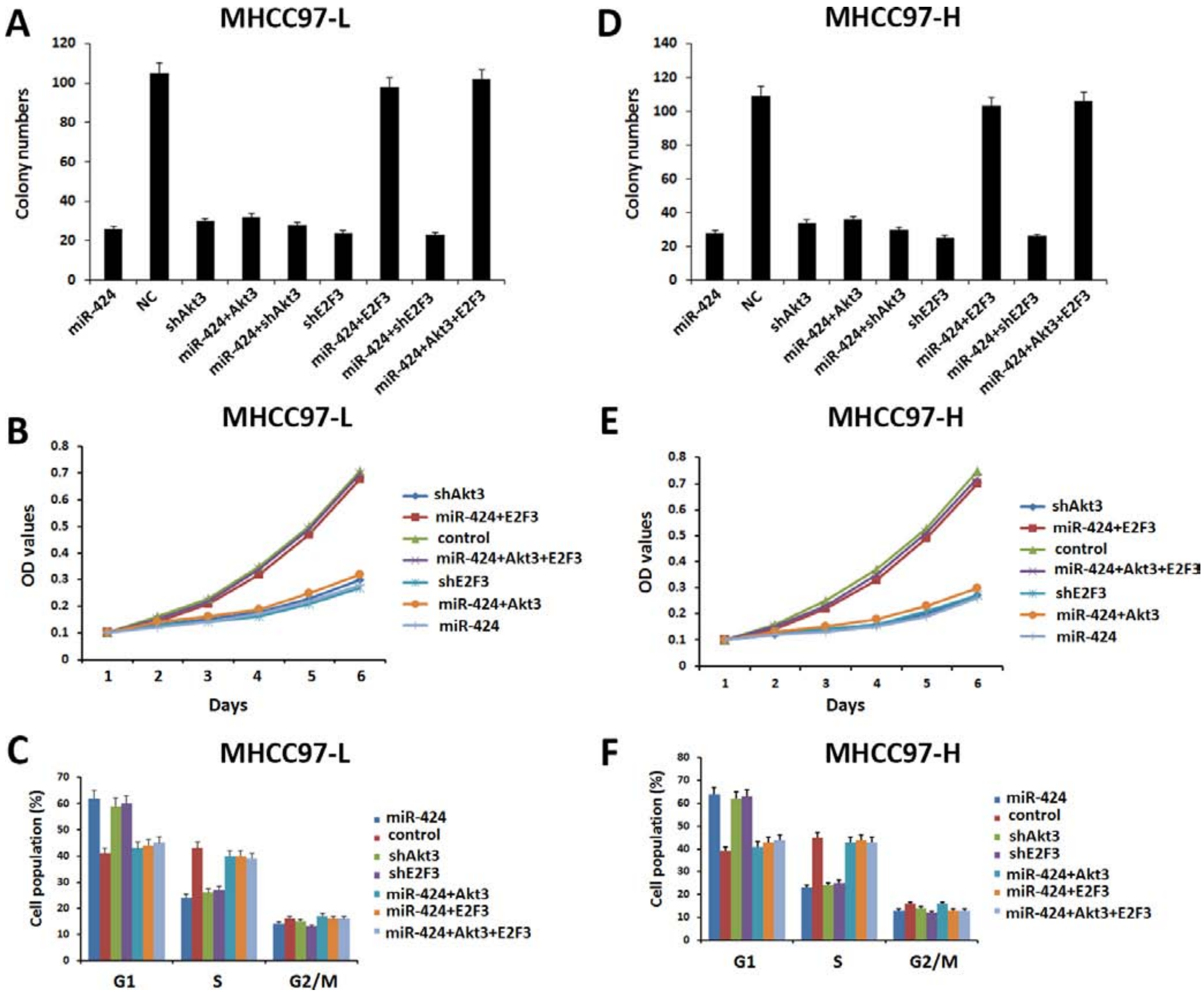

Supplementary Figure S6: The clonogenic assay, cell proliferation and cell cycle assay in MHCC97-L A–C. and MHCC97-H cell lines D–F.

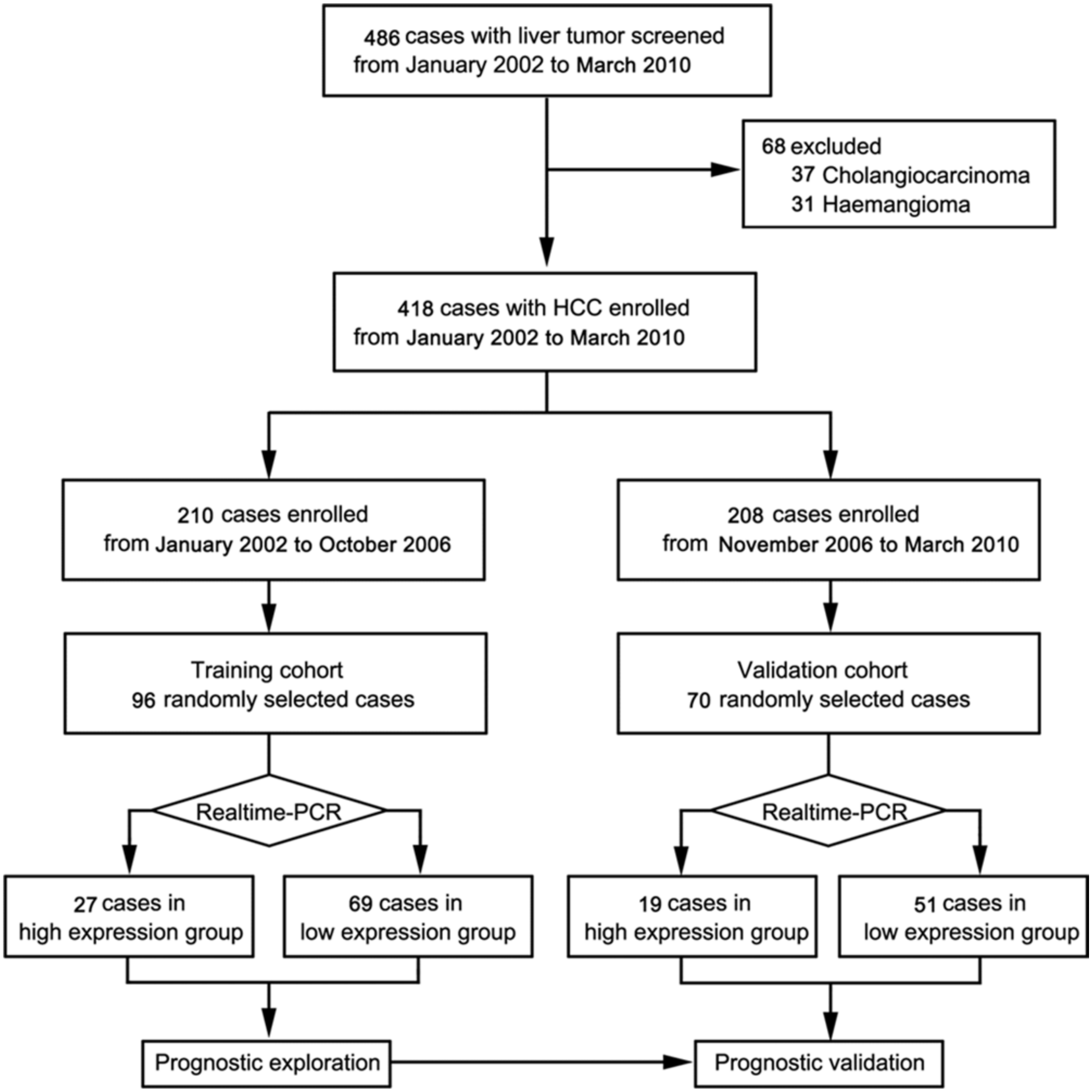

Supplementary Figure S8: Diagrammatic sketch of patients enrolled and groups designed.

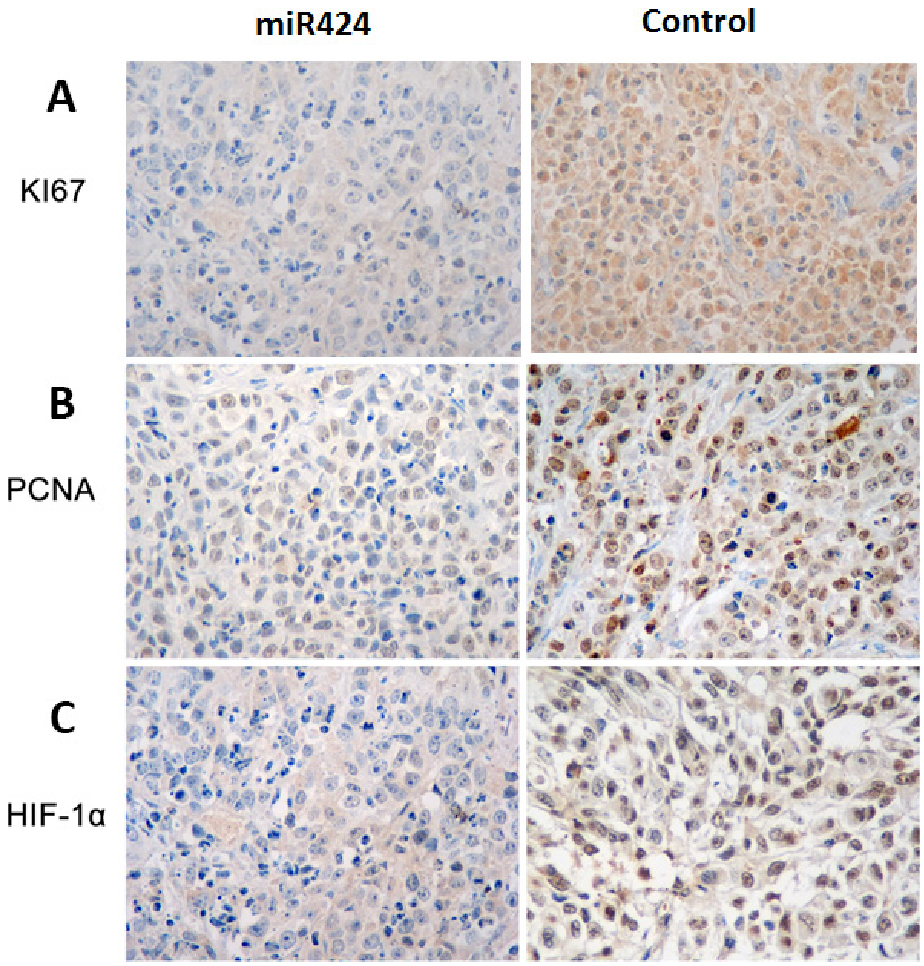

Supplementary Figure S9: IHC for Ki67 A. PCNA B. and HIF-1 alpha were performed in miR-424 overexpressed group mice tumor and control group mice tumor.

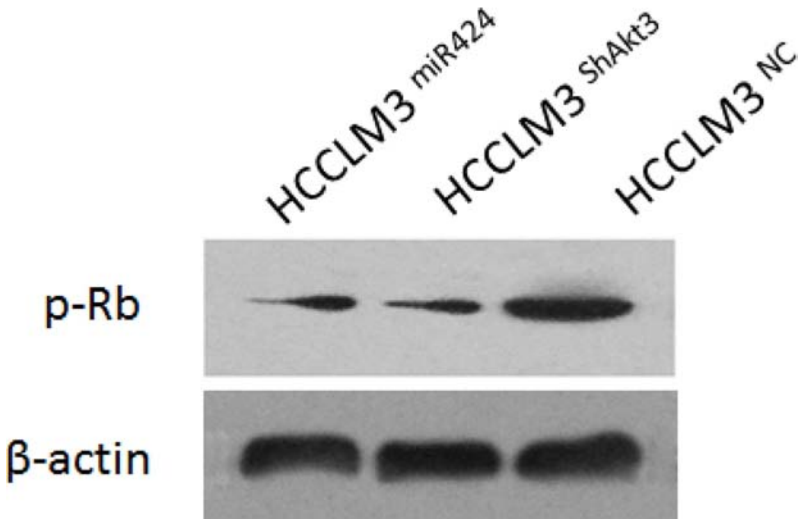

Supplementary Figure S10: WB for p-Rb were performed in HCCLM3$^{miR-424}$, HCCLM3^{NC} and HCCLM3^{shAkt3} cells.

Supplementary Table S1: miR-424 expression was inversely correlated with Ki-67 expression.

	N	miR-424		*R* value	*P* value
		Low expression	High expression		
Ki-67 expression					
Positive	84	72	12		
Negative	12	4	8	−0.7583	0.007

Supplementary Table S2. Correlations between miR-424 expression level and clinicopathological variables of 70 cases of HCC in validation cohort

Clinicopathologic Variables	*N*	miR-424 expression		*P* value
		Low	High	
Gender				
Male	58	44	14	
Female	12	7	5	0.286
Age(years)				
≤ 60	45	32	13	
> 60	25	19	6	0.782
HBsAg				
Negative	21	16	5	
Positive	49	35	14	0.776
AFP				
Negative	8	4	4	
Positive	62	47	15	0.200
Cirrhosis				
Absence	26	17	9	
Presence	44	34	10	0.404
Child-Pugh Score				
A	23	14	9	
B	47	37	10	0.154
Tumor size (cm)				
≤5	20	8	12	
>5	50	43	7	**<0.001**
Capsular formation				
Presence	32	25	7	
Absence	38	26	12	0.426
Tumor nodule number				
Solitary	47	38	9	
Multiple(≥2)	23	13	10	**0.045**
TNM Stage				
I/II	55	44	11	
III	15	7	8	**0.019**
BCLC Stage				
0-A	48	40	8	
B-C	22	11	11	**0.008**

(*Continued*)

Clinicopathologic Variables	*N*	miR-424 expression		*P* value
		Low	High	
Edmondson-Steiner Stage				
I-II	24	17	7	
III-IV	46	34	12	0.784
Vein invasion				
Presence	12	8	4	
Absence	58	43	15	0.723

Supplementary Table S3. The Cox regression analyses of Disease-free survival (DFS) and miR-424 expression level as well as clinicopathological parameters in training cohort

	Univariable analysis		Multivariable analysis	
**Variables**	**HR (95% CI)**	***P***	**HR (95% CI)**	***P***
Gender(Male *vs.* Female)	1.082 (0.472 − 2.524)	0.632		
Age(≤60 *vs.* >60)	0.628 (0.398 − 3.142)	0.423		
HBsAg(Negative *vs.* Positive)	0.875 (0.512 − 2.926)	0.714		
Cirrhosis(Presence *vs.* Absence)	1.875 (1.142 − 6.472)	**0.036**	1.708 (0.898 – 4.824)	0.058
Child-Pugh Score(Grade A *vs.* B)	0.816 (0.432 − 2.022)	0.667		
AFP level(ng/ml, ≤20 *vs.* >20)	0.673 (0.247 − 1.821)	0.396		
Tumor size(>5 *vs.* ≤5 cm)	1.784 (0.768 − 2.764)	0.196		
Capsular formation(Presence *vs.* Absence)	0.576 (0.324 − 1.081)	0.072	0.708 (0.432 – 1.555)	0.654
Tumor nodule number(Solitary *vs.* Multiple)	0.538 (0.308 − 0.944)	**0.032**	0.478 (0.284 – 0.956)	**0.025**
Edmondson-Steiner Stage(I-II *vs.* III-IV)	0.592 (0.391 − 1.393)	0.283		
Vein invasion(Presence *vs.* Absence)	1.966 (1.312 − 3.104)	**0.012**	1.988(1.142 – 3.428)	**0.016**
miR-424 expression(Low *vs.* High)	2.188 (1.322 − 4.772)	**0.008**	2.452 (1.245 – 4.828)	**0.022**

Supplementary Table S4. The Cox regression analyses of overall survival (OS) and miR-424 expression level as well as clinicopathological parameters in validation cohort

	Univariable analysis		Multivariable analysis	
**Variables**	**HR (95% CI)**	***P***	**HR (95% CI)**	***P***
Gender(Male *vs.* Female)	0.884 (0.423 − 2.356)	0.456		
Age(≤60 *vs.* >60)	0.798 (0.456 − 2.132)	0.587		
HBsAg(Negative *vs.* Positive)	1.145 (0.576 − 2.323)	0.689		
Cirrhosis(Presence *vs.* Absence)	2.475 (1.323 − 8.432)	**0.019**	1.712 (0.813 – 4.583)	0.312
Child-Pugh Score(Grade A *vs.* B)	1.453 (0.632 − 2.431)	0.785		
AFP level(ng/ml, ≤20 *vs.* >20)	0.673 (0.274 − 1.782)	0.352		
Tumor size(>5 *vs.* ≤5 cm)	1.897 (0.876 − 2.872)	0.098	3.235 (0.942 – 3.678)	0.176
Capsular formation(Presence *vs.* Absence)	1.463 (0.554 − 3.462)	0.332		
Tumor nodule number(Solitary *vs.* Multiple)	3.731 (1.142 − 8.994)	**0.025**	4.246 (1.513 – 9.216)	**0.008**
Edmondson-Steiner Stage(I-II *vs.* III-IV)	1.156 (0.747 − 1.836)	0.435		
Vein invasion(Presence *vs.* Absence)	1.746 (1.384 − 3.438)	**0.032**	3.613 (1.085 – 8.282)	**0.023**
miR-424 expression(Low *vs.* High)	2.312 (1.350 − 7.172)	**0.009**	3.152 (1.065 – 8.278)	**0.016**

Supplementary Table S5. The Cox regression analyses of Disease-free survival (DFS) and miR-424 expression level as well as clinicopathological parameters in validation cohort

	Univariable analysis		Multivariable analysis	
**Variables**	**HR (95% CI)**	***P***	**HR (95% CI)**	***P***
Gender(Male *vs.* Female)	1.113 (0.345 − 2.242)	0.586		
Age(≤60 *vs.* >60)	0.586 (0.366 − 2.984)	0.512		
HBsAg(Negative *vs.* Positive)	0.892 (0.484 − 3.025)	0.778		
Cirrhosis(Presence *vs.* Absence)	1.682 (1. 413 − 5.765)	**0.038**	1.842 (1.218 – 6.412)	**0.040**
Child-Pugh Score(Grade A *vs.* B)	0.778 (0.398 − 2.664)	0.712		
AFP level(ng/ml, ≤20 *vs.* >20)	0.613 (0.318 − 2.115)	0.452		
Tumor size(>5 *vs.* ≤5 cm)	1.886 (0.653 − 3.221)	0.214		
Capsular formation(Presence *vs.* Absence)	0.665 (0.412 − 1.724)	0.189		
Tumor nodule number(Solitary *vs.* Multiple)	0.682 (0.499 − 0.985)	**0.027**	0.522 (0.312 – 0.965)	**0.025**
Edmondson-Steiner Stage(I-II *vs.* III-IV)	0.615 (0.317 − 1.265)	0.189		
Vein invasion(Presence *vs.* Absence)	1.856 (1.134 − 4.032)	**0.010**	2.012(1.088 – 3.892)	**0.014**
miR-424 expression(Low *vs.* High)	2.221 (1.112 − 5.231)	**0.002**	2.334 (1.422 – 6.241)	**0.006**

Supplementary Table S6. Clinicopathologcal characteristics of the patients

Clinicopathologic Variables	*N*	*N*
	**Training cohort**	**Validation cohort**
Median age (range)	50.5 years (22–78)	52.5 years (20–81)
Gender		
Male	80	58
Female	16	12
HBsAg		
Negative	29	21
Positive	67	49
AFP		
Negative	11	8
Positive	85	62
Cirrhosis		
Absence	36	26
Presence	60	44
Child-Pugh Score		
A	31	23
B	65	47
Tumor size (cm)		
≤5	28	20
>5	68	50
Capsular formation		
Presence	44	32
Absence	52	38
Tumor nodule number		
Solitary	64	47
Multiple(≥2)	32	23
TNM Stage		
I/II	76	55
III	20	15
BCLC Stage		
0-A	66	48
B-C	30	22
Edmondson-Steiner Stage		
I-II	33	24
III-IV	63	46
Vein invasion		
Presence	17	12
Absence	79	58
